# Prevalence of IgG Autoantibodies against GD3 Ganglioside in Acute Zika Virus Infection

**DOI:** 10.3389/fmed.2018.00025

**Published:** 2018-03-09

**Authors:** Dirlei Nico, Luciana Conde, Juan L. Rivera-Correa, Andréia Vasconcelos-dos-Santos, Louise Mesentier-Louro, Leonardo Freire-de-Lima, Mônica Barcellos Arruda, Celio Geraldo Freire-de-Lima, Orlando da Costa Ferreira, Maria Elisabeth Lopes Moreira, Andrea Araújo Zin, Zilton Farias Meira Vasconcelos, Rosalia Mendez Otero, Clarisa Beatriz Palatnik-de-Sousa, Amilcar Tanuri, Adriane Regina Todeschini, Wilson Savino, Ana Rodriguez, Alexandre Morrot

**Affiliations:** ^1^Instituto de Microbiologia Paulo de Góes, Universidade Federal do Rio de Janeiro, Rio de Janeiro, Brazil; ^2^Department of Microbiology, New York University School of Medicine, New York, NY, United States; ^3^Instituto de Biofísica Carlos Chagas Filho, Universidade Federal do Rio de Janeiro, Rio de Janeiro, Brazil; ^4^Departamento de Genética, Universidade Federal do Rio de Janeiro, Rio de Janeiro, Brazil; ^5^Instituto Nacional de Saúde da Mulher, da Criança e do Adolescente Fernandes Figueira, Unidade de Pesquisa Clínica, Instituto Oswaldo Cruz, Fiocruz, Rio de Janeiro, Brazil; ^6^Instituto Oswaldo Cruz, Fiocruz, Rio de Janeiro, Brazil; ^7^National Institute of Science and Technology on Neuroimmunomodulation (INCT-NIM), Rio de Janeiro, Brazil; ^8^Faculdade de Medicina, Centro de Pesquisas em Tuberculose, Universidade Federal do Rio de Janeiro, Rio de Janeiro, Brazil; ^9^Instituto Oswaldo Cruz, Laboratório de Imunopatologia, FIOCRUZ, Rio de Janeiro, Brazil

**Keywords:** Zika virus, gangliosides, autoimmunity, autoantibody, infectious diseases

## Abstract

Zika virus (ZIKV) disease has become a global health emergency with devastating effects on public health. Recent evidences implicate the virus as an emergent neuropathological agent promoting serious pathologies of the human nervous system, that include destructive and malformation consequences such as development of ocular and fetal brain lesions, microcephaly in neonates, and Guillain–Barré syndrome (GBS) in adults. These neurological disorders of both central and peripheral nervous systems are thought to be associated to the neurotropic properties of the virus that has ability to infect neural stem cells as well as peripheral neurons, a hallmark of its pathogenicity. The presence of autoantibodies against gangliosides plays a pivotal role in the etiogenesis of GBS and a variety of neurological disorders. Gangliosides are a class of galactose-containing cerebrosides mainly expressed in nervous system tissues playing a critical role in the physiology of neural cells and neurogenesis. Herein, our findings indicate that patients at acute phase of ZIKV infection without any neurological signs show increased levels of IgG autoantibody against GD3 gangliosides, a class of glycolipid found to be highly expressed in neural stem cell acting in the maintenance of their self-renewal cellular capacity. It is possible that a pathological threshold of these antibodies is only acquired in secondary or subsequent infections. In the light of these evidences, we propose that the target of GD3 by autoimmune responses may possibly has an effect in the neuropathy and neurogenesis disorder seen during ZIKV infection.

## Introduction

Zika virus (ZIKV) disease has become a global health emergency with devastating effects on public health. The ZIKV is a positive-sense, single-stranded RNA *Flavivirus* transmitted to humans primarily by infected mosquitoes (1). Recently, studies have shown an association of ZIKV infection and the development of Guillain–Barré syndrome (GBS), a disorder of peripheral nervous system generated by autoimmune responses (2). The syndrome, usually associated with either motor axonal neuropathy or inflammatory demyelinating polyneuropathy, occurs due to previous infection with *Campylobacter jejuni*, cytomegalovirus, Epstein–Barr virus (EBV), *Mycoplasma pneumonia, Haemophilus influenza*, or influenza A virus. However, *C. jejuni*, cytomegalovirus, and EBV prevail as preceding infectious pathogens. All these pathogens have carbohydrate antigen sequences in common with peripheral nerve tissue, and their infections are associated with anti-ganglioside antibodies (3).

Molecular mimicry between microbial and nerve glycolipid antigens has been characterized in GBS after *Campylobacter* infections and is thought to be a major driving force behind the development of the disorder (3). However, the origin of autoimmunity in GBS after ZIKV infections is still unclear, since anti-glycolipid antibodies were only found in a fraction of the patients and no antibody competition was found between sphingolipid GA1 and ZIKV proteins (2). Autoimmune responses targeting gangliosides may contribute to the neurological complications related to ZIKV infection (4). The infection is associated with severe neurological manifestations of microcephaly associated with disease.

Zika virus has been detected in amniotic fluid of pregnant women whose fetuses had been diagnosed with microcephaly, suggesting that ZIKV can cross the placental barrier (5). In experimental mice models, studies have shown a vertical transmission of ZIKV in mice with infection of radial glia cells from dorsal ventricular zone of the fetuses, affecting the brain development (6). In addition, independent studies have demonstrated that ZIKV infection attenuates growth of human neural progenitor cells (7). Furthermore, other studies have determined the effects of ZIKV on cultured human neural progenitors or 3D organoids, derived from induced pluripotent stem cells, demonstrating that the virus specifically infect neural progenitors and inhibited their proliferation (8).

However, the mechanistic insight concerning the basis of ZIKV-induced neuropathogenesis is unknown. In this study, we sought to characterize this response evaluating the presence of IgG autoantibodies against gangliosides in ZIKV infection. Gangliosides are cell-membrane structures of lipophilic sphingolipids containing a sialylated carbohydrate chains involved in cell-to-cell recognition, playing pivotal roles in biological processes including cell motility, growth, and tissue differentiation (9, 10). These glycolipids are the major glycans of the vertebrate brain, representing up to 12% of membrane lipid of the gray matter in human brain. They are concentrated in large quantities in the ganglion cells of the central nervous system (CNS) (10). Since the gangliosides are crucial in neurological development and their expression correlates with neurogenesis (10), autoimmune responses targeting the gangliosides may represent an underexploited opportunity to examine the increased incidence of neurological complications related to ZIKV infection.

## Materials and Methods

### Ethics Statement

This study was approved by the Research Ethics Committee of Research Institute Prof. Joaquim Amorim Neto (IPSEP), Campina Grande, Brazil (protocol #52888616.4.0000.5693). Protocols for human studies were approved by the Institutional Ethical Committees in accordance with international guidelines. All individuals provided written informed consent. Protocols for animal handling and experimental procedures were approved by the Research Ethics Committee of Federal University of Rio de Janeiro (CEUA protocol #064). All animal experimentation protocols were performed in accordance with the terms of the Brazilian guidelines for the animal welfare regulations followed the guidelines set by the National Institutes of Health, United States.

### Study Population

This study was approved by the Research Ethics Committee of Gaffrée and Guinle University Hospital, Rio de Janeiro, Brazil (CAAE#54448216.2.0000.5258) and the Research Ethics Committee of Fernandes Figueira Institute, FIOCRUZ, Rio de Janeiro, Brazil (CAAE#52675616.0.0000.5269) in accordance with international guidelines. Healthy volunteers were recruited from Gaffrée and Guinle University Hospital and Zika-infected patients were recruited from Fernandes Figueira Institute. None of the Zika-infected patients presented autoimmune pathologies. All individuals provided written informed consent. Subjects using any medication that could affect immune functions were excluded from the study. Sex- and age-matched controls were also included. The diagnosis was based on clinical signs and symptoms including mild fever, skin rash, conjunctivitis, muscle and joint pain (Tables 1 and 2). All infected patients and non-infected control individuals were screened for Zika polymerase chain reaction (RT-PCR) to confirm the diagnosis for virus infection using one-step RT-PCR for the detection of the envelope-protein coding region (350 bp) from blood and plasma samples. Briefly, we used a set of primers targeting ZIKV envelope with a sensitivity of 25 copies per reaction [ZIKV forward (1,086–1,102) CCGCTGCCCAACACAAG; ZIKV env reverse (1,162–1,139) CCACTAACGTTCTTTTGCAGACAT; and ZIKV probe (1,107–1,137) FAM—AGCCTACCTTGACAAGCAGTCAGACACTCAA–BHQ1]. The PCR was run using a positive containing 100–200 copies/ml of ZIKV genome and a negative control. ELISA to detect the presence of IgM antibody against ZIKV from serum samples obtained from infected patients and non-infected control individuals was performed using IgM antibody capture Zika MAC-ELISA standardized by the Centers for Disease Control and Prevention (CDC, Fort Collins, CO, USA) in accordance with manufacturer protocol (11). Briefly, plates were coated with 75 µl of goat anti-human IgM (Kirkegaard and Perry Laboratories, USA) in carbonate/bicarbonate buffer (pH 9.6) and incubated overnight at 4°C. Blocking was done with PBS (pH 7.2), 5% non-fat dry-milk/0.05% tween 20, for 30 min at room temperature (RT) before the washing step. Afterward, 50 µl of serum samples diluted at 1/400 in PBS pH 7.2, 0.05% tween 20, and negative (pooled *Flavivirus*-negative serum) or positive (CDC humanized 6B6C-1 pan-*Flavivirus*) controls were added before incubation at 37°C for 1 h. Viral Zika antigen (CDC Vero E6 derived, inactivated ZIKV antigen) or normal control antigen (CDC Vero E6 derived, mock-infected normal antigen) were used (50 µl) as controls. The immunocomplexes were detected by the addition of horseradish peroxidase-conjugated secondary antibody (6B6C-1, CDC) diluted in blocking buffer for 1 h at 37°C. Subsequently, the reaction was developed by the addition of TMB, and after 10 min incubation at RT, the reaction was stopped with 1 N sulfuric acid solution and the optical density (OD) was read at 450 nm. The ratio (P/N) was calculated as follows: mean OD of the test sample reacted on viral antigen (P) divided by the mean OD of the negative control reacted on viral antigen (N). P/N value <3.0 was negative; >3.0 was positive.

**Table 1:** Clinical data of adult Zika patients whose serum samples were included in the study.

Samples	Gender	Symptoms onset (date)	Zyka PCR	Zika exposure
A#1	M	02/21/2016	+	Y
A#2	M	03/10/2016	+	Y
A#3	F	Unknown	+	Y
A#4	M	03/25/2016	+	Y
A#5	F	02/05/2016	+	Y
A#6	M	Unknown	+	Y
A#7	F	04/17/2016	+	Y
A#8	F	04/27/2016	+	Y
A#9	F	04/25/2016	+	Y
A#10	F	05/01/2016	+	Y
A#11	F	05/03/2016	+	Y
A#12	M	Unknown	+	Y
A#13	M	Unknown	+	Y

**Table 2:** Clinical data of pregnant patients with Zika infection whose serum samples were included in the study.

Samples	Pregnancy period (symptoms onset)	Gestacional age (weeks)	Zyka PCR	Zyka serology (IgM)	Zika exposure
P#1	3rd trimester	38	+	Not done	Y
P#2	1st trimester	39	+	Not done	Y
P#3	1st trimester	38	−	+	Y
P#4	1st trimester	41	+	Not done	Y
P#5	1st trimester	39	+	Not done	Y
P#6	3rd trimester	38	+	Not done	Y
P#7	2nd trimester	38	−	+	Y
P#8	1st trimester	40	+	Not done	Y
P#9	3rd trimester	40	+	Not done	Y
P#10	2nd trimester	38	+	Not done	Y
P#11	1st trimester	39	+	Not done	Y
P#12	2nd trimester	38	−	+	Y

### Animals and Histological Preparations

GD3 synthase null mice, genetic 129/SvEv background, generated by Kawai et al. (12) was a gift from Dr. Steven Walkley (Department of Neuroscience, Albert Einstein College of Medicine). The knockout mice and their counterpart wild-type animals were maintained in the experimental animal facility from Biophysics Institute at the Federal University of Rio de Janeiro. Animal handling and experimental protocols were approved by the Animal Care and Use Committee of the Institution (CEUA protocol #064). Adult (3- to 5-month-old) mice were used in this study, and all surgical procedures were performed under anesthesia with isoflurane. For the immunohistochemical assays using retina sections, animals receiving an overdose of isoflurane were perfused through the heart with ice-cold saline, followed by 4% paraformaldehyde in 0.1 M phosphate buffer, pH 7.4. Following the removal of the eyes, the tissues were post-fixed in 4% paraformaldehyde for 2 h at 8–10°C and then transferred to increasing sucrose solutions until 30%. The samples were embedded in optimal cutting temperature (Tissue-Tek) and sectioned longitudinally at 14 µm thickness using a cryostat (Leica Microsystems, Wetzlar, Germany). Tissue sections, stored at −20°C, were rinsed with 0.01% Triton X-100 in PBS (PBST) and incubated in blocking solution (5% normal goat serum, Sigma-Aldrich Co) for 30 min at RT, followed by incubation with peripheral blood serum of either Zika patients or healthy donors (1:50 non-pregnant; 1:25 pregnant) and primary control antibody (CD60b, 1:50) overnight at 4°C. Sections were then washed in PBST and incubated with 1:1,500 secondary antibodies (goat IgG anti-human FITC-conjugated and goat IgG anti-mouse IgMμ Cy3-conjugated, Jackson Laboratories) and 1:1,500 TO-PRO-3 iodide (Invitrogen Inc.) for 2 h at RT. Afterward, slides were rinsed in PBS and mounted with coverslips using 0.01% (w/v) p-phenylenediamine in 90% glycerol and then analyzed under a confocal microscope (Zeiss LSM 510 Meta). Images were acquired using a 63× magnification objective lens.

### Anti-ganglioside ELISA Assay

Total and GD3 gangliosides were purchased from Avanti Polar lipids (13) with purity >99%. GT1b, GD1a, GD1b, GM2, and GT1b gangliosides were purchased from Matreya, LLC (PA, USA) with purity >98%. All gangliosides were resuspended in a organic solution of chloroform:methanol as a one-time use stock and coated at 20 μg/ml in 200 proof molecular biology ethanol and allowed to evaporate at RT after 16 h of coating at 4 C. Calf thymus DNA (Sigma) was coated 100 μg/ml in sterile PBS for 16 h at 4 C. After antigen coating, the plates were incubated with blocking buffer (milk 10%, 0.05% tween 20, PBS) during 2 h at RT. The antibody levels were assayed in sera from normal individuals and Zika-infected patients. We use secondary antibodies such as HRP-conjugated mouse anti-human IgG1, IgG2, IgG3, IgG4, or total IgG (Invitrogen, made in USA) in a 1:2,000 dilution in blocking buffer. The reaction was developed with *O*-phenyldiamine (Sigma) or TMB (BD Bioscience), interrupted with 1 N sulfuric acid, and monitored at 492 or 450 nm, respectively (BioRad). Each individual serum was analyzed by triplicates or duplicates. Results were expressed as the mean of the absorbance values (492 nm) of the 1/100 diluted sera of each patient. Controls for the GD3 ELISA including assay specificity, GD3 plate coating, assay conditions, and selection of the optimal sera dilution to be tested in the ELISA were performed (Figure S1 in Supplementary Material).

### Statistical Analysis

Results were expressed as mean ± SE, unless otherwise indicated. Parametric and non-parametric tests were used, depending on the characteristics of variables (normal distribution or not, etc). Comparisons among groups were made by parametric analysis of variance (k groups > 2) followed by Student’s *t*-test (k groups = 2) or non-parametric Kruskall–Wallis test (k groups > 2) followed by the Mann–Whitney *U*-test (k groups = 2). Differences between control versus infected groups were considered statistically significant when *p* ≤ 0.05.

## Results

**Figure 1:**
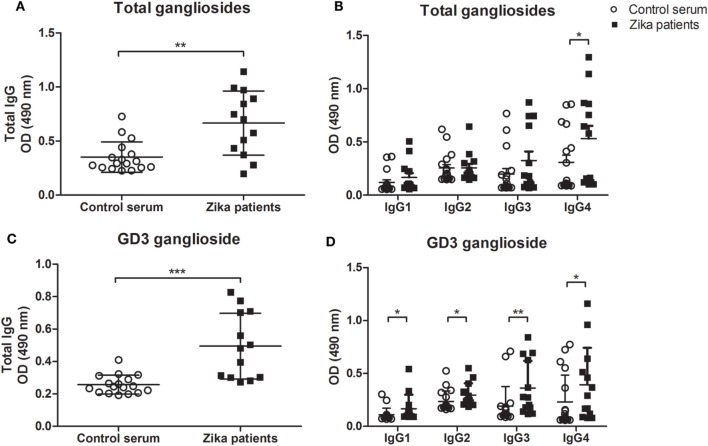
Autoantibody levels to gangliosides in Zika-infected patients and healthy control individuals. ELISA plates coated with the indicated gangliosides at 20 µg/ml in ethanol, followed by evaporation, were incubated with 1:100 dilution of sera from Zika patients or control healthy individuals and developed with anti-total IgG or anti-Ig-specific subclasses conjugated to HRP. Scatter plots show individual values for each Zika-infected patient (*n* = 13) and healthy individual (*n* = 17) for recognition of **(A,C)** IgG class autoantibodies and **(B,D)** IgG subclasses (IgG1, IgG2, IgG3, and IgG4 isotypes), against **(A,B)** total gangliosides (from bovine brain extract) or **(C,D)** purified ganglioside GD3. Each data point is the mean of triplicated determinations. Means of data points for each individual ± SE are shown. Differences between groups are significant *(*p* ≤ 0.05), **(*p* ≤ 0.01), ***(*p* ≤ 0.01).

To determine whether anti-ganglioside antibodies are present in patients with Zika infection, we tested the serum for IgG autoantibodies against an extract of brain gangliosides by ELISA. We found a significant increase in the levels of total IgG autoantibodies recognizing brain gangliosides in the sera of Zika-infected patients (Figure 1A). This difference may be the contribution to a particular isotype, as our data indicated higher frequencies of IgG4 anti-gangliosides in infected patients as compared to healthy control individuals (Figure 1B). Next, we screened the sera of all patients and healthy controls for IgG subclasses against five human brain gangliosides. We tested compounds belonging to a diverse group of gangliosides that are abundant in nervous tissues, namely, GD1a, GD3, GM2, GD1b, and GT1b (10).

The members GD1a, GM2 contain one sialic acid molecule linear to the fatty acyl sphingosine (ceramide) structure. The GD1b, GT1b, and GD3 gangliosides have a branched sugar chain containing two sialic acid molecules attached to the lipid core (10). We found no significant differences in the frequencies of autoantibodies against GT1b, GD1b, GD1a, and GM2 gangliosides between the study groups (Figure S2 in Supplementary Material). However, our data indicate elevated levels of IgG autoantibodies against ganglioside GD3 in Zika-infected patients as compared healthy individuals (Figure 1C). This increased level of anti-GD3 autoantibody was not linked to any specific IgG isotype (Figure 1D). Autoreactivity to DNA, a common feature of many autoimmune and infection-related responses (14) was also tested, showed no differences between Zika patients and controls for IgG subclasses, except for IgG4 (Figure S3 in Supplementary Material).

**Figure 2:**
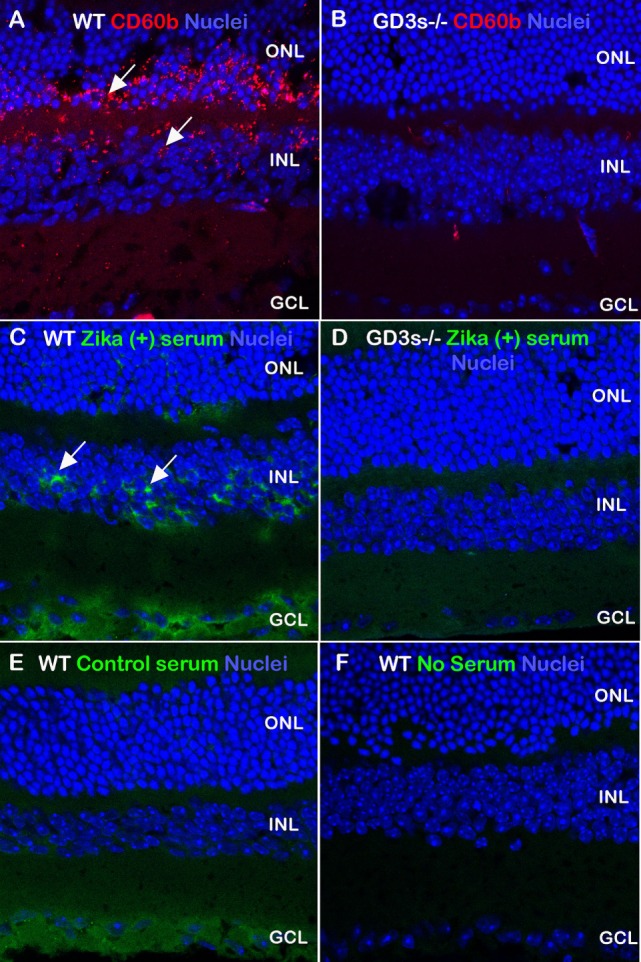
Autoantibody profile in Zika-infected patients recognizing GD3 gangliosides from retinal tissue. Retinal sections obtained from wild-type and GD3-deficient mice were stained with control antibody (CD60b, red) to detect the 9-*O*-acetylated form of GD3 ganglioside. Serum derived from Zika patients **(A,C,E)** recognized antigens (green) in the wild-type retina, more abundantly in the inner nuclear layer (arrows). Control healthy serum **(B,D,F)** did not recognize antigens in the outer and inner nuclear layers in which GD3 is largely expressed. A level of serum reactivity for the ganglion cell layer was noticed in wild-type sections exposed to both human sera groups. Images are merged with nuclei (TOPRO, blue).

Next, we investigated whether the specificity of anti-GD3 autoantibodies present in the serum of patients from acute phase of infection is in fact capable of recognizing the ganglioside expressed in its tissue conformation. To approach this issue, we tested the specificity of this autoantibody response using immunohistochemistry in retinal tissues of wild-type and GD3-deficient mice as controls. Gangliosides are conserved structurally among mammals, and neurons from retina contain the prevalence of GD3, a ganglioside distinctive of proliferative neural cells (15–17). According to our results, the expression of GD3 ganglioside in the adult retina was observed in the wild-type mice as a bright punctiform pattern throughout the outer and inner nuclear layers when we used sample serum with high anti-GD3 antibody titles from Zika-infected patients as compared healthy individuals (Figure 2). This pattern was also obtained when we assessed the expression of 9-*O*-acetylated form of GD3 in wild-type retina using the CD60b antibody as positive control (Figure 2).

**Figure 3:**
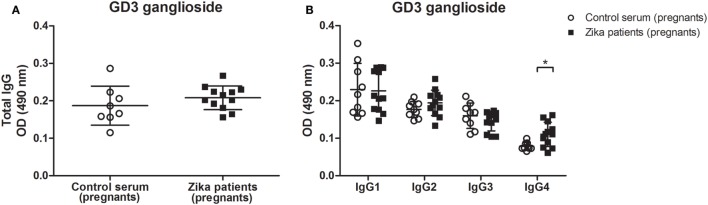
Increased levels of IgG4 autoantibody against to GD3 ganglioside in Zika virus infection during pregnancy. ELISA plates coated with GD3 ganglioside (20 µg/ml) were incubated with 1:100 dilution of sera from pregnant patients with Zika infection or control healthy pregnant individuals. The sera was obtained after pregnancy, and the reaction was developed with **(A)** anti-total IgG or **(B)** anti-Ig-specific subclasses conjugated to HRP. Scatter plots show individual values for each Zika-infected patient (*n* = 12) and healthy individual (*n* = 8). Means of data points for each individual ± SE are shown. Differences between groups are significant *(*p* ≤ 0.05).

By contrast, the retinal immunostaining pattern seen for GD3 ganglioside was not observed when CD60b antibody was incubated with tissue sections from the GD3-deficient mouse, or the serum from control healthy patients were incubated with the retinal section of wild-type mouse (Figure 2). Some labeling in the ganglion cell layer has been noticed in wild-type sections exposed to both human sera groups, but not in the retina sections obtained from GD3-deficient mouse (Figure 2). This pattern of self-reactivity to GD3 of the antibodies from patients may have a significant impact on the pathophysiology of disease during the pregnancy (5, 18). In fact, our data generated from the ELISA method indicated that IgG4 isotype anti-GD3 ganglioside autoantibodies are significantly increased in pregnant patients with Zika infection as compared to healthy pregnant controls (Figure 3).

## Discussion

The growing number of serious neurological complications associated with ZIKV led the World Health Organization to declare a public health emergency of international concerns, which is having devastating effects on public health in epidemic regions (19). ZIKV has been isolated from newborn tissues as well as amniotic fluid from pregnant women indicating a maternofetal transmission (5). It has been shown that ZIKV can cross the placental barrier causing development of brain abnormalities in newborns (5, 6, 20–23). Although the microcephaly has been the most common reported clinical manifestation in newborns whose mothers acquired Zika virus during pregnancy, anencephaly cases are also associated with infection at earlier stages of fetal development (21). These malformation sequels induced by infection might be associated with the ability of virus to infect neural stem cells thus harming the neurogenesis of neural tissues and organs and might be related to the stages of pregnancy affected at the time of infection (7, 8). In adults, Zika virus infection is associated with ascending paralysis and myelitis (5, 24), although the pathophysiology of these clinical manifestations is unknown. Infection in animal models has demonstrated a direct effect of ZIKV in the pathology of disease (25).

This study shows that Zika-infected patients at acute phase, even if they do not present autoimmune disorders, frequently develop low levels of autoreactive antibodies against gangliosides and in particular against GD3. Low titers of these antibodies are also found in patients with uncomplicated *C. jejuni* infections without neurological pathology associated, suggesting that autoreactivity is a frequent reaction to both infections, but autoimmune pathology is only developed in a minority of individuals (26). The low titers of anti-GD3 autoantibodies may be due to the time of exposure of patients to infection. However, it is not clear that a higher viral load during an infection would correlate with higher autoimmunity, since autoreactivity is also influenced by host factors that are independent of the viral load. It is possible that the low titers of autoantibodies against ganglioside GD3 are due to a regulatory T cell-mediated control that may vary among patients (27). In fact, evidence show that a Treg/Th17 imbalance plays a critical role in the neuropathogenesis of Zika infection (28). In addition, studies aiming at characterizing the neuro-immune interaction mechanisms underlying the neurovirulence of infection show the involvement of CD8 T cell-mediated neuropathology autoimmune responses in susceptible murine experimental models, suggesting blood–barrier disruption in the development of neurological complications associated with virus infection (29).

Our data indicate that there is not a specific subclass of anti-GD3 antibodies developed, although a significance difference is found for IgG4 in pregnant acutely infected patients. Previous findings in GBS show that anti-GM1 and anti-GT1a antibodies are predominantly IgG1 and IgG3 (30) and anti-GQ1b can either be IgG2 or IgG3, depending on the clinical manifestation (31). However, it has been described that the antibody response to GD3 in general is made of different subclasses of antibodies (32), these data are in line with our findings for adult acutely infected individuals. The demonstration that the infection yields increased levels of IgG4 during pregnancy is relevant since IgG4 autoimmune antibodies against different antigens are found in various autoimmune diseases and neurological disorders, including GBS (33, 34). Although the disease mechanism of GBS after ZIKV infection may be very different than in GBS after other infections, our study shows an increase in total ganglioside and anti-GD3 antibodies in Zika patients and points out that it may be related to the broad neuropathy associated with the disease (35).

The induction of autoantibodies against GD3 in fact might have a profound disturbance in the biology of neural cells as this ganglioside can mediate several cellular responses such as proliferation, differentiation, and apoptosis (36–38). The binding of autoantibodies to GD3 ganglioside may lead to the homeostatic imbalance of neural cells, thus affecting the neurogenesis during embryonic/fetal development. These findings are important in view of the fact that neurological complications and CNS abnormalities have been reported in congenital infections in Zika patients (5, 6, 20–23). The congenital Zika syndrome is also associated with retinal lesions of newborns (16, 21). Since GD3 ganglioside plays an important role during retina development (15, 16), our findings demonstrating the targeting of acquired autoantibodies against this molecule by Zika-infected pregnant patients could be determined in the pathogenesis of disease. Although our studies have only detected low levels of autoantibodies against GD3 in acutely infected adults and pregnant patients, it is possible that a pathological threshold of these antibodies is only acquired in secondary or subsequent infections.

Moreover, it has been shown that GD3 ganglioside interacts with EGF receptor to sustain the self-renewal ability of neural stem cells (39). The autoantibody recognition of GD3 could inhibit this interaction or alternatively trigger programmed cell death pathways thus leading an impaired neural function. In addition, it has been shown that GD3 ganglioside regulates the proinflammatory response of microglia induced by interleukin-15 (IL-15). The binding of ganglioside to IL-15 inhibits the IL-15-dependent T-cell proliferation as well as the production of nitric oxide and nuclear factor-kappaB activity (40). Since microglial cells play a major role in the inflammatory events from CNS, the binding of autoantibodies to GD3 may prevent the interaction of this ganglioside to IL-15 thus potentiating the inflammatory processes and outcome of CNS tissue damage during ZIKV infection.

These findings are relevant since GD3 gangliosides are found to be expressed in neural human and mouse stem cells (17, 41). Although it is still not confirmed whether neural stem cells are infected in Zika patients, these cells are infected by ZIKV *in vitro* (8) and in mice (42). It is possible that during viral shedding from infected neural stem cells expressing GD3 gangliosides, ZIKV obtains the lipid molecules from the cell membrane during the viral budding process. The recognition of GD3 autoantigen by the immune system in the context of pathogen-associated molecular patterns would lead to a break of peripheral tolerance to GD3 ganglioside thus resulting in autoantibody production and increasing the likelihood of developing self-destructive autoimmune disease (43).

Autoantibodies against GD3 might induce a profound disturbance in the biology of neural cells as this ganglioside can mediate several cellular responses such as proliferation (39), differentiation (44), and apoptosis (45). Our findings suggest that GD3 could mediate the neuropathologies that are associated with Zika infections and that this ganglioside might be developed as a biomarker to identify patients at risk of developing autoimmune complications in the context of ZIKV infections. Furthermore, prospective multidisciplinary follow up of patients in secondary or subsequent infections will allow a better understanding of the pathological threshold of autoantibodies against GD3 ganglioside in ZIKV infection.

## Ethics Statement

This study was approved by the Research Ethics Committee of Research Institute Prof. Joaquim Amorim Neto (IPSEP), Campina Grande, Brazil (protocol #52888616.4.0000.5693). Protocols for human studies were approved by the Institutional Ethical Committees in accordance with international guidelines. All individuals provided written informed consent.

## Author Contributions

DN, LC, JR-C, AV-d-S, CA, LM-L, and MA conducted the experiments; DN, LC, CA, LM-L, and MA acquired data; DN, LC, CA, LM-L, OF, MA, and AM analyzed data; CP-d-S, AT, WS, AR, and AM designed research studies; LF-d-L, CF-d-L, OF, MM, AZ, ZV, RO, CP-d-S, AT, ART, WS, AR, and AM provided reagents; OF, ZV, AT, WS, AR, and AM wrote the manuscript. All the authors have read and approved the final manuscript.

## Conflict of Interest Statement

The authors declare that the research was conducted in the absence of any commercial or financial relationships that could be construed as a potential conflict of interest.

## Supplementary Material

Figure S1 ELISA for the detection of anti-GD3 antibodies. ELISA plates were coated with GD3 and incubated with serial dilutions of Zika–infected samples, control uninfected samples, or an anti-GD3 monoclonal antibody as positive control. Zika-infected and control samples were incubated in plates at 4°C or at room temperature followed by washes with PBS alone or with 0.05% tween-20, respectively.

Figure S2 Zika virus infection does not increase the frequency to GD1a, GD1b, GM2, and GT1b anti-ganglioside antibodies. ELISA plates coated with different gangliosides were incubated with 1:50 dilution of sera from Zika patients or control healthy individuals. Scatter plot showing individual values for each patient (*n* = 4) and normal individuals (*n* = 4). Data are mean ± SE and represent the results of three independent experiments.

Figure S3 Anti-DNA antibody responses in Zika patients and health control individuals. ELISA plates coated with calf thymus DNA were incubated with 1:50 dilution of sera from Zika patients or control healthy individuals. Scatter plot showing individual values for each patient (*n* = 7) and normal individuals (*n* = 7). Data are mean ± SE and represent the results of three independent experiments. Differences between groups are significant *(*p* ≤ 0.05).
