# Supplementary figures and images for: Prevalence of IgG Autoantibodies against GD3 Ganglioside in Acute Zika Virus Infection
Source: Front Med (Lausanne). 2018 Mar 9;5:25. doi: 10.3389/fmed.2018.00025 (PMC5854646; doi:10.3389/fmed.2018.00025)

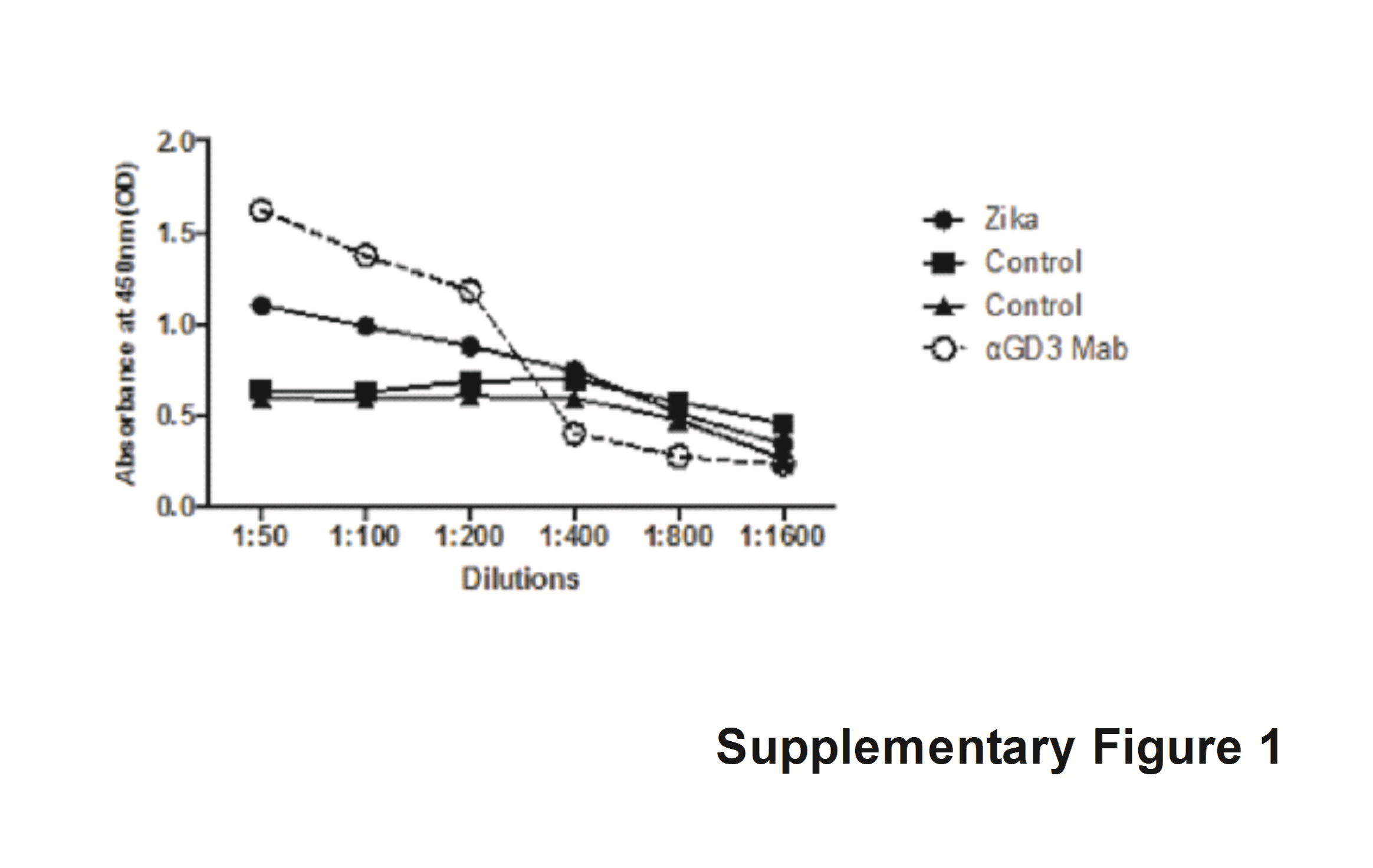

Supplement: Figure S1 — ELISA for the detection of anti-GD3 antibodies. ELISA plates were coated with GD3 and incubated with serial dilutions of Zika–infected samples, control uninfected samples, or an anti-GD3 monoclonal antibody as positive control. Zika-infected and control samples were incubated in plates at 4°C or at room temperature followed by washes with PBS alone or with 0.05% tween-20, respectively. [file Image_1.tif]

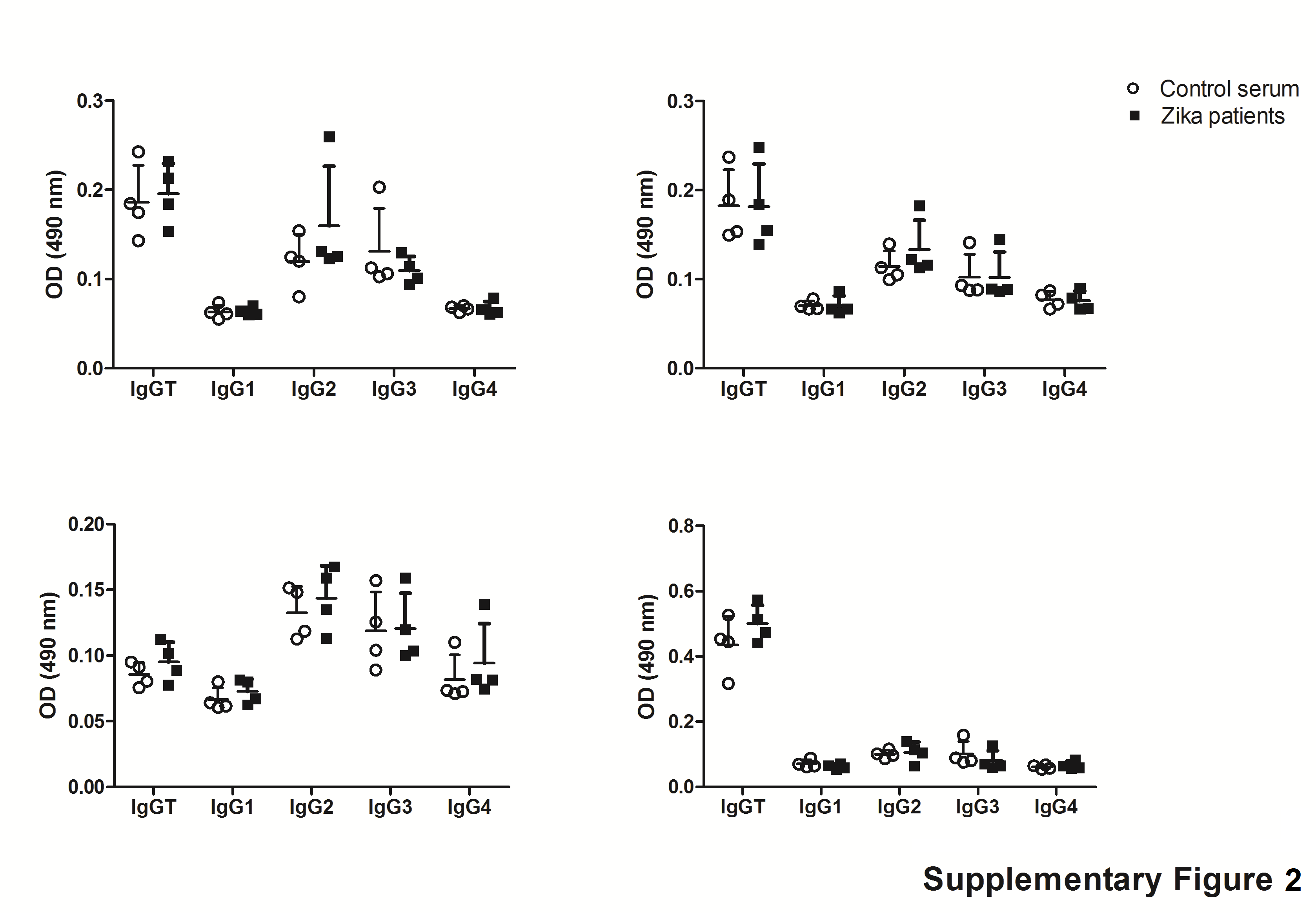

Supplement: Figure S2 — Zika virus infection does not increase the frequency to GD1a, GD1b, GM2, and GT1b anti-ganglioside antibodies. ELISA plates coated with different gangliosides were incubated with 1:50 dilution of sera from Zika patients or control healthy individuals. Scatter plot showing individual values for each patient (n = 4) and normal individuals (n = 4). Data are mean ± SE and represent the results of three independent experiments. [file Image_2.tif]

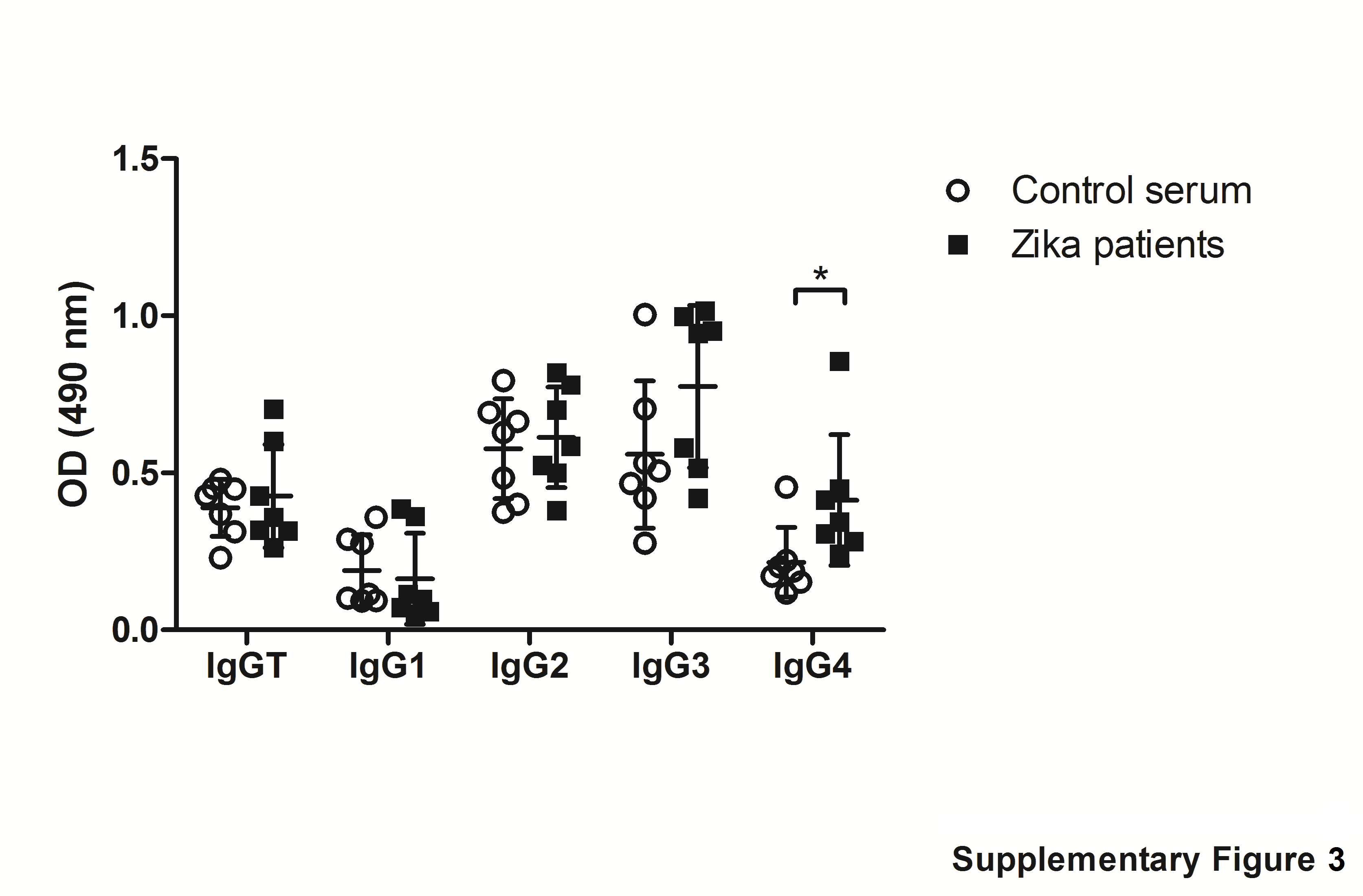

Supplement: Figure S3 — Anti-DNA antibody responses in Zika patients and health control individuals. ELISA plates coated with calf thymus DNA were incubated with 1:50 dilution of sera from Zika patients or control healthy individuals. Scatter plot showing individual values for each patient (n = 7) and normal individuals (n = 7). Data are mean ± SE and represent the results of three independent experiments. Differences between groups are significant *(p ≤ 0.05). [file Image_3.tif]
